# Dumping Syndrome and Bile Acid Reflux Following Pyloroplasty and Gastric Peroral Endoscopic Myotomy (G-POEM) for Refractory Gastroparesis: A Systematic Review

**DOI:** 10.7759/cureus.79056

**Published:** 2025-02-15

**Authors:** Ryan Shargo, Isaac Poonen-Honig, Camille Thélin, Christopher G DuCoin, Rahul Mhaskar, Salvatore Docimo, Joseph A Sujka

**Affiliations:** 1 Department of Medical Education, University of South Florida Morsani College of Medicine, Tampa, USA; 2 Department of Gastroenterology, University of South Florida Morsani College of Medicine, Tampa, USA; 3 Department of Surgery, University of South Florida Morsani College of Medicine, Tampa, USA

**Keywords:** bile acid gastritis, bile acid reflux, dumping syndrome, gastric peroral endoscopic myotomy, gastroparesis treatment, gpoem, pyloroplasty

## Abstract

Treatment options for medication- and diet-resistant gastroparesis include drainage procedures such as pyloroplasty and gastric peroral endoscopic myotomy (G-POEM). While dumping syndrome (DS) and bile acid gastritis (BAG) have been documented as complications following pyloric drainage procedures, limited evidence exists concerning their incidence after pyloroplasty and G-POEM for refractory gastroparesis. We performed a systematic review of outcomes following pyloroplasty and G-POEM for refractory gastroparesis (PROSPERO: CRD42024559654). PubMed, Embase, Cochrane, and Web of Science databases were systematically searched for articles reporting rates of DS and BAG following pyloric drainage procedures. Assessed outcomes included rates of DS and BAG. Results were narratively synthesized and presented descriptively. Of the 2278 records reviewed, 10 studies were included. Six studies evaluated pyloroplasty, and four studies evaluated G-POEM. Nine studies reported incidence of DS, and two studies reported rates of BAG. The incidence of DS ranged from 0% to 23.1%, with a median incidence of 3.23% and an interquartile range of 6.95% among nine studies. The incidence of BAG ranged from 0% to 15.4% in two studies. The studies displayed high heterogeneity in study design, patient population, and co-interventions, precluding collective data analysis and limiting generalizability. All studies displayed a moderate to serious risk of bias. There exists a paucity of data regarding the incidence of DS and BAG following pyloroplasty and G-POEM for refractory gastroparesis. Despite the theoretical association of these complications with pylorus-modifying procedures, our findings suggest that they may be uncommon or underreported in the treatment of refractory gastroparesis. Improved reporting of these outcomes and robust prospective studies investigating the incidence of such complications are required.

## Introduction and background

Gastroparesis is a chronic dysmotility disorder characterized by delayed gastric emptying without mechanical obstruction [1]. The etiology of gastroparesis is diverse, with diabetes mellitus being the most common cause, followed by idiopathic origins and post-surgical complications, including following vagotomy. Symptoms include nausea, vomiting, early satiety, bloating, and abdominal pain, which can significantly impact the quality of life and nutritional status of affected individuals [2]. Diagnosis of gastroparesis is commonly confirmed through a four-hour gastric emptying study known as gastric emptying scintigraphy. The test consists of a radiolabeled meal that is traced through the upper gastrointestinal tract through serial imaging at different time points [2,3]. A retention rate greater than 10% of the study meal four hours after consumption is indicative of gastroparesis [3]. Gastric emptying scintigraphy has previously been shown to have a 44% sensitivity and 93% specificity for the diagnosis of gastroparesis [4].

Gastroparesis treatment options start with dietary modifications and medications, eventually progressing to more invasive interventions as necessary. Dietary changes often involve small, frequent meals that are low in fat and fiber to facilitate gastric emptying [2]. Medications such as prokinetic agents (e.g., metoclopramide and domperidone), antiemetics (e.g., ondansetron), and pain modulators (e.g., tricyclic antidepressants) are commonly prescribed to manage symptoms [5]. However, dietary changes are rarely sufficient alone, and prokinetic agents have become less favored clinically due to their adverse safety profiles [6].

In cases of gastroparesis refractory to dietary and pharmacologic intervention, gastric electrical stimulation (GES) has also been utilized. This involves the implantation of a neurostimulator device to deliver electrical pulses to modulate the interstitial cells of Cajal [7]. GES has shown several positive clinical outcomes for the treatment of gastroparesis. A study by Heckert et al. found that 75% of patients experience symptom improvement following neurostimulator placement, with 43% showing at least moderate improvement, particularly in nausea, loss of appetite, and early satiety [8]. However, device complications are seen in about 15% of patients, with a removal rate of 6.3-12.8% [9].

Pyloroplasty and gastric peroral endoscopic myotomy (G-POEM) represent surgical alternatives for the management of gastroparesis, particularly in cases refractory to conservative management. Rates of surgical intervention for gastroparesis vary, with Gray et al. finding that 5.53% of patients treated in urban teaching hospitals undergo surgical intervention, compared to 3.94% in urban non-teaching hospitals and 2.38% in rural hospitals [10]. Open and laparoscopic pyloroplasty involve the creation of a longitudinal incision in the pylorus to facilitate gastric emptying. Such procedures have been shown to be efficacious for patients with refractory gastroparesis, with 86% of patients showing improved gastric emptying on scintigraphy and 77% achieving normalization [11]. In 2013, Khashab et al. demonstrated G-POEM in a human patient for the first time, introducing the procedure as a less invasive alternative to pyloroplasty with lower rates of post-procedural complications and a shorter post-surgical hospital stay [12,13]. These interventions aim to expand the pyloric opening and diminish its sphincter functionality, operating on the principle that an enlarged pylorus should expedite gastric emptying. Contraindications to such procedures include confirmed mechanical gastric outlet obstruction as determined by esophagogastroduodenoscopy, opioid dependence without prior weaning and reassessment of gastric emptying, and severe bleeding risk [14].

While pyloroplasty and G-POEM offer significant benefits, complications arise in about 11% of cases [14]. Adverse events include manageable pain, typically treated with analgesics; bleeding, which may necessitate endoscopic procedures or blood transfusions; and bowel perforation, a critical condition requiring prompt surgical intervention. Additional complications are mucosotomy, often resolved through endoscopic clipping; pneumoperitoneum and capnoperitoneum, usually managed conservatively but occasionally requiring intervention; and infections, addressed with antibiotics and potentially surgical drainage for severe cases [15]. Other complications include rapid emptying of gastric contents, leading to dumping syndrome (DS) or reflux of the contents of the duodenum back into the antrum, causing bile acid gastritis (BAG) [16,17]. Few studies report these complications in patient cohorts following pyloric interventions, particularly as they are utilized for the treatment of refractory gastroparesis [18-21]. Most evidence comes from case reports [22,23] or theoretical background [16,17]. There is currently no population-based data regarding the incidence of DS and BAG following such procedures for gastroparesis [18]. This study aims to conduct a systematic review of the literature regarding the incidence of DS and BAG following both pyloroplasty and G-POEM to better understand their rate of occurrence after these procedures.

## Review

Methods

A systematic review was performed according to a predefined protocol (registered on PROSPERO: CRD42024559654) and reported according to the Preferred Reporting Items for Systematic Reviews and Meta-Analyses (PRISMA) guidelines [24].

Search Strategy and Selection Criteria

A comprehensive search protocol was devised to identify research on outcomes of pyloroplasty and G-POEM for refractory gastroparesis (Appendix S1). On June 1, 2024, a search was conducted across PubMed, Embase, Cochrane, and Web of Science databases. This was further enhanced by manually examining citations and reference lists and tracking citations of relevant publications throughout the review process. The search was limited to English-language articles. All studies reporting on pyloroplasty or G-POEM for refractory gastroparesis were reviewed for inclusion. However, the final selection focused on those studies reporting on the adverse events of DS and BAG. Studies that examined these complications were included, even if they were ultimately found to not occur in the study cohort. Studies with pediatric samples were excluded. Letters to the editor, abstracts without full texts, commentaries, editorials, case reports, and case series were excluded per our PROSPERO registration protocol.

Study Selection and Data Extraction

Database search results were extracted and deduplicated. Subsequently, title and abstract screening was performed by two independent reviewers (RS and IPH) on Covidence (Veritas Health Innovation Ltd., Melbourne, Australia) [25], with conflicts resolved by consensus. Articles were then sought for full-text access and review. The two independent reviewers performed a full-text review with conflicts for inclusion decisions resolved by consensus. Microsoft Excel (Microsoft Corporation, Redmond, Washington) was utilized to extract study characteristics (author, year of publication, country, study design, treatment/experimental groups, participant characteristics, type of intervention, length of follow-up, and rates of DS and BAG). Data were extracted by an independent reviewer (IPH), with another reviewer (RS) assessing data for accuracy and rectifying inconsistencies.

Risk of Bias Assessment

For quality evaluation, information regarding study design and methods was collected. The assessment utilized the Cochrane Risk of Bias in Non-Randomized Studies of Interventions (ROBINS-I) tool [26]. This tool evaluates seven distinct domains of potential bias: confounding, selection of participants, classification of interventions, deviations from intended interventions, missing data, measurement of outcomes, and selection of reported results. Based on these criteria, each study was categorized as having either an overall low, moderate, or serious risk of bias.

Data Analysis

Data were narratively synthesized and presented descriptively as frequencies and percentages. The incidence of DS and BAG was extracted from each included study. Median incidence rates and interquartile ranges for DS and BAG were computed. Studies were compared based on criteria for diagnosis and follow-up durations of patients who developed DS and BAG. Additionally, the context of each study was analyzed, including patient demographics, previous surgical history, and the type of surgical intervention (pyloroplasty vs. G-POEM).

Results

Study Selection

**Figure 1:**
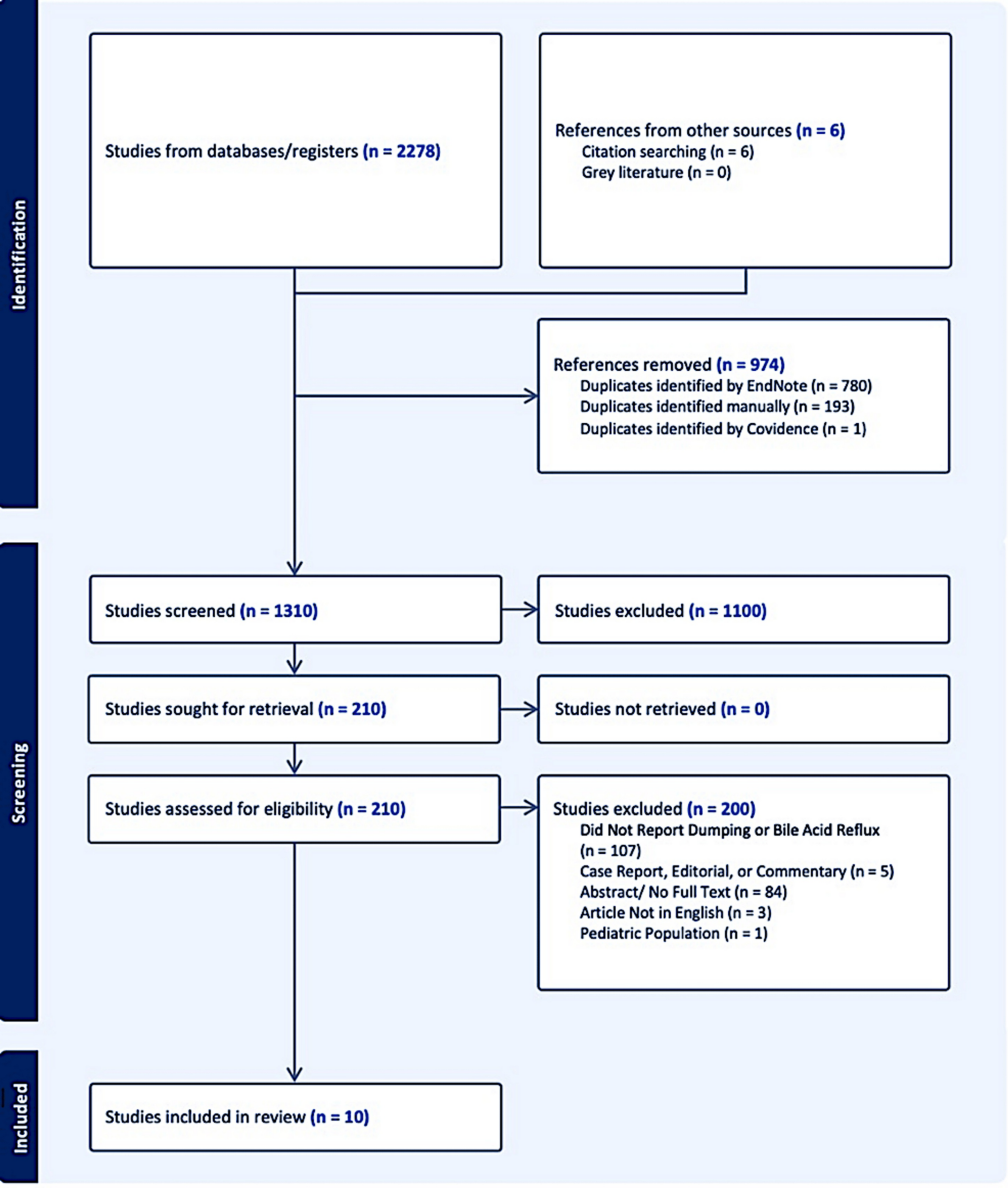
PRISMA flow diagram for article identification, screening, and inclusion PRISMA: Preferred Reporting Items for Systematic Reviews and Meta-Analyses Adapted from Page et al. [24]

Overall, 2278 records were identified from database searching, of which 974 duplicates were removed. Following title and abstract screening, 210 articles were sought for retrieval based on their mention of pyloroplasty or G-POEM for refractory gastroparesis. Of these, 107 full texts were excluded for not mentioning DS or BAG, 84 records were excluded for lacking a full text, five records were excluded as they were commentaries, case reports, or editorials, three records were excluded for not being in English, and one article was excluded for investigating a pediatric population. This yielded 10 articles for final inclusion. A full flow diagram assessing study identification, screening, and inclusion is presented in Figure 1.

Study Characteristics

This systematic review included 10 studies published between 2012 and 2022. Most of the studies (seven out of 10) were conducted in the United States of America. The remaining studies were international, with one conducted across multiple international sites, one in the Czech Republic and Slovakia [27], and one in France [28]. The study designs varied, with six retrospective single-arm studies, two prospective studies, one randomized controlled trial, and one prospective study with a comparator. The results of each study are extracted in Table 1.

**Table 1:** Study characteristics This table presents the study characteristics of the studies identified through the systematic search protocol. Studies without a specific characteristic are noted as not reported (NR) in the associated cell. BAG = bile acid gastritis; GES = gastric electrical stimulation; G-POEM = gastric peroral endoscopic myotomy

Study	Country	Study Design	Intervention	Patient Characteristics	Patients in Treatment Arm	Patients With Dumping	Patients With BAG	Follow-up (Months)
Sarosiek et al. 2013 [29]	USA	Prospective with Comparator	Open Pyloroplasty with GES vs. GES alone	13 previously undergone truncal vagotomy. 17 Diabetic; 23 Post-Surgical; 9 Idiopathic	26	2	NR	7
Toro et al. 2014 [30]	USA	Retrospective Single Arm	Laparoscopic Pyloroplasty	34 patients (68%) had previous foregut procedures and/or cholecystectomy	50	0	NR	3
Datta et al. 2014 [31]	USA	Retrospective Single Arm	Open Pyloroplasty	Prior esophagectomy and refractory to endoscopic therapy	13	3	2	12
Mancini et al. 2015 [32]	USA	Retrospective Single Arm	42 Laparoscopic Pyloroplasty; 4 Open Pyloroplasty	None with previous gastric surgery or GES insertion. 15 Diabetic; 31 Idiopathic	46	0	NR	12
Davis et al. 2017 [33]	USA	Retrospective Single Arm	Open Pyloroplasty + GES	17 Diabetic; 10 Idiopathic	24	2	NR	38
Wellington et al. 2020 [34]	USA	Prospective	Laparoscopic Pyloroplasty	3 Diabetic; 7 Idiopathic	10	1	NR	6
Ichkhanian et al. 2021 [35]	International Multicenter	Retrospective Single Arm	G-POEM	Patients experiencing adverse events. 15 Diabetic; 4 Post-Surgical; 12 Idiopathic	31	1	NR	2–4 weeks
Ichkhanian et al. 2022 [36]	USA Multicenter	Retrospective Single Arm	G-POEM	De novo gastroparesis post lung transplant	20	NR	0	8.9
Martinek et al. 2022 [27]	Czech Republic and Slovakia	Randomized Control Trial	G-POEM vs. Sham Procedure	12 patients crossed over to G-POEM. 9 Diabetic; 6 Post-Surgical; 6 Idiopathic	32	1	NR	6
Baret et al. 2022 [28]	French Multicenter	Retrospective Single Arm	G-POEM	64 Diabetic; 53 Post-Surgical; 83 Idiopathic; 9 Scleroderma; 9 Other	217	3	NR	3–6 months

Six studies focused on various forms of pyloroplasty, open and laparoscopic. These studies were all conducted in the USA between 2012 and 2020. The sample sizes ranged from 10 to 50 patients, with a total of 169 patients across all pyloroplasty studies. The interventions varied, including open pyloroplasty, laparoscopic pyloroplasty, and combination with GES. The follow-up periods ranged from three to 38 months. The incidence of DS ranged from 0% [30,32] to 23.1% [31].

Four studies focused on G-POEM. These were more recent, published between 2020 and 2022, and had a more international representation. The G-POEM studies included a total of 300 patients, with individual study sample sizes ranging from 20 to 217 patients. Three of these were retrospective single-arm studies, while one was a randomized controlled trial comparing G-POEM to a sham procedure [27]. The follow-up periods for G-POEM studies were generally shorter, ranging from two to four weeks to 8.9 months, with one study reporting a three- to six-month follow-up [28]. The incidence of DS ranged from 1.38% [28] to 3.23% [35].

Nine studies reported the incidence of DS in their patient population for a total of 13 patients. Two studies defined DS as less than 30% of the study meal remaining at one hour and less than 20% remaining at two hours during gastric emptying scintigraphy [29,33]. Wellington et al. defined DS solely by emptying criteria at one hour [34]. The remainder of the studies did not define the criteria for DS. The etiology of gastroparesis for patients developing DS was reported for five patients, with all patients presenting with post-surgical gastroparesis. Of these, two patients had undergone prior truncal vagotomy [29], and three patients had previous esophagectomy [33]. Overall, the incidence of DS ranged from 0% to 23.1%, with a median incidence of 3.23% and an interquartile range of 6.95%.

Treatment, timing, and course of DS were reported in seven patients. Ichkhanian et al. reported one patient developed DS within the first 48 hours following G-POEM, presenting with episodes of hypoglycemia accompanied by symptoms of tachycardia and sweating [35]. The patient was managed with an IV dextrose solution, reporting the resolution of symptoms with dietary modification. Martinek et al. reported one patient to develop DS three months following G-POEM [27]. The patient developed hypoglycemia, requiring 30 days of hospitalization without mention of specific interventions. Baret et al. reported one patient developed DS in the early postoperative period, and two developed DS in the late postoperative period, up to one month [28]. All three patients presented with hypoglycemia, requiring hospitalization and improving within five days. The authors described a favorable course without recurrence after the introduction of dietary measures to avoid major glycemic peaks [28]. Davis et al. reported a diagnosis of DS in two patients at 38 months but did not comment on the course of treatment [33].

Incidence of BAG was reported in two studies, with Datta et al. reporting BAG in two patients [31] and Ichkhanian et al. reporting no patients to have developed BAG [36]. Of four patients with postoperative complications following open pyloroplasty, three patients developed DS, and two patients developed BAG [31]. BAG was endoscopically proven with evidence of reflux esophagitis that required medical therapy. At least one patient developed both DS and BAG. The authors did not indicate the timing of such complications or the course of medical therapy.

Risk of Bias

Of the 10 studies selected for inclusion, the risk of bias was determined to be serious for five and moderate for the remainder. Accounting for confounding was low, with five (50%) studies failing to account for potential confounders, being classified as a serious risk of bias in confounding (Domain 1). The remaining five studies failed to account for unavoidable confounders, being classified as a moderate risk of bias in confounding (Domain 1). Only one randomized trial was included [27]. Most of the studies had a low risk of bias in their selection of participants as outlined in Domain 2 (90%), classification of interventions as outlined in Domain 3 (100%), and deviations from the intended intervention as outlined in Domain 4 (90%). Table 2 provides a detailed risk of bias assessment, including all domains assessed.

**Table 2:** Risk of bias in non-randomized studies regarding exposure assessment This table presents the results of the risk of bias assessment utilizing the Cochrane Risk of Bias in Non-Randomized Studies of Interventions (ROBINS-I) tool. The studies were evaluated for risk of bias in confounding (D1), selection of participants (D2), classification of interventions (D3), deviations from intended interventions (D4), missing data (D5), measurement of outcomes (D6), selection of reported results (D7), and overall risk of bias.

Study	D1	D2	D3	D4	D5	D6	D7	Overall
Sarosiek et al. 2013 [29]	Serious	Low	Low	Low	Low	Moderate	Low	Serious
Toro et al. 2014 [30]	Moderate	Low	Low	Moderate	Low	Low	Moderate	Moderate
Datta et al. 2014 [31]	Serious	Low	Low	Low	Low	Serious	Moderate	Serious
Mancini et al. 2015 [32]	Moderate	Low	Low	Low	Moderate	Moderate	Moderate	Moderate
Davis et al. 2017 [33]	Moderate	Low	Low	Low	Moderate	Moderate	Low	Moderate
Wellington et al. 2020 [34]	Serious	Low	Low	Low	Low	Moderate	Moderate	Serious
Ichkhanian et al. 2021 [35]	Moderate	Low	Low	Low	Low	Low	Low	Moderate
Ichkhanian et al. 2022 [36]	Serious	Low	Low	Low	Moderate	Low	Moderate	Serious
Martinek et al. 2022 [27]	Moderate	Moderate	Low	Low	Low	Low	Low	Moderate
Baret et al. 2022 [28]	Severe	Low	Low	Low	Low	Low	Low	Severe

Discussion

Incidence of DS and BAG

This systematic review aimed to evaluate the incidence of DS and BAG following pyloroplasty and G-POEM in patients with refractory gastroparesis. A total of 10 studies met the inclusion criteria, including six studies evaluating pyloroplasty and four studies evaluating G-POEM. Of these, nine studies reported the incidence of DS, and two studies reported rates of BAG. The incidence of DS ranged from 0% to 23.1%, with a median incidence of 3.23% and an interquartile range of 6.95%. The incidence of BAG ranged from 0% to 15.4%.

Impact of Prior Surgery, Concurrent Procedures, and Patient Population

Interestingly, many of the included studies evaluated patient populations with prior surgical history or simultaneous interventions. This complicates the accurate description of rates of dumping and BAG, as prior or concurrent interventions may influence patient presentation. For example, in the study by Sarosiek et al. [29], 13 of 26 patients in the surgical pyloroplasty group had previously undergone truncal vagotomy, often performed for the treatment of peptic ulcer disease [37]. Notably, these patients did not receive a subsequent drainage procedure, resulting in post-surgical gastroparesis that necessitated pyloroplasty. Both patients diagnosed with DS in this group had a history of vagotomy [29]. This historical factor is significant as vagotomy, especially without a drainage procedure, is known to predispose patients to DS due to changes in gastric motility and the subsequent rapid emptying of gastric contents [38]. This rapid transit can lead to both early and late DS symptoms, complicating the interpretation of DS and BAG rates post-pyloroplasty. Furthermore, these patients also received concurrent GES, a treatment whose mechanism is hypothesized to modulate gastrointestinal motility but has been shown not to accelerate gastric emptying [39].

Toro et al. examined a cohort where 68% of patients had undergone a prior foregut procedure and/or cholecystectomy, yet none of the 50 patients exhibited DS [30]. Such foregut surgeries are known to increase the risk of DS, and this risk escalates further when combined with cholecystectomy [40]. Similarly, Datta et al. [31] studied patients who had all undergone esophagectomy, a procedure linked to both DS [41] and BAG [42], and who were refractory to endoscopic therapy. These patients had multiple interventions, including an average of 3.4 ± 1.0 endoscopic balloon dilations, and seven out of 13 patients also received intrapyloric botulinum toxin injections [31]. Despite the established connections between these procedures and DS, the patient groups in studies by Sarosiek et al., Toro et al., and Datta et al. all presented with gastroparesis prior to undergoing pyloroplasty. Given that DS involves rapid gastric emptying, in contrast to the delayed emptying characteristic of gastroparesis, the induction of rapid emptying in these cases was likely a direct consequence of the pyloroplasty. However, as DS and BAG can occur independently of gastroparesis, the presence of prior or simultaneous interventions significantly complicates the interpretation of their relationship and the outcomes observed.

Other studies aimed to provide a comprehensive analysis of adverse events in patients with refractory gastroparesis undergoing G-POEM. However, they include patient cohorts that may not be representative of all patients with refractory gastroparesis receiving pyloroplasty or G-POEM. In 2020, Ichkhanian et al. specifically examined adverse events following G-POEM, matching each patient with an adverse event to two controls [35]. The authors found that one of 31 (3.23%) patients with adverse events experienced DS. However, this population does not provide information regarding the risk for all patients undergoing G-POEM. Additionally, in 2022, Ichkhanian et al. examined patients with refractory gastroparesis post lung transplant and found no patients to develop BAG [36]. It is important to note that none of the included studies aimed to determine incidence rates of DS or BAG following pyloroplasty or G-POEM and thus are expected to present confounding when trying to generalize their results.

Trends in the Diagnosis of DS and BAG

Of the studies that reported DS and BAG as a complication of pyloroplasty or G-POEM, similar trends in diagnosis and presentation emerge. Three studies reported that the criteria for diagnosis of DS included less than 30% of the study meal remaining at one hour during gastric emptying scintigraphy. This criterion is recommended by a 2008 consensus statement by the American Neurogastroenterology and Motility Society and the Society of Nuclear Medicine [43]. Other methods to diagnose DS include oral glucose tolerance testing (OGTT), with a more recent 2020 international consensus reporting OGTT as the preferred method of DS diagnosis [44]. It is unclear whether other studies reporting rates of DS utilized gastric emptying scintigraphy or OGTT in the diagnosis of DS. BAG was reported in one study by Datta et al. and was diagnosed endoscopically [31].

Information regarding the etiology of gastroparesis for patients who developed DS was available for five patients. Interestingly, all five patients had initially presented with post-surgical gastroparesis, either following truncal vagotomy or esophagectomy. While this may be purely coincidental, given the small sample, it may be possible that prior surgical loss of vagal innervation may contribute to the inability to adequately regulate gastric emptying after pyloroplasty. Similarly, both patients who developed BAG had previously been diagnosed with post-surgical gastroparesis.

The timing of the presentation of DS varied among patients, ranging from 48 hours to 38 months in seven patients. Four patients were diagnosed with DS within one month of intervention, with one patient diagnosed at three months and two patients at 38 months postoperatively. Information regarding presenting symptoms was available for six of these patients, with all patients presenting with hypoglycemia. The variability in the timing of presentation may be due to variations in follow-up between study protocols. Additionally, DS may have manifested with differing degrees of severity in patients, leading some to seek care in the acute postoperative period while others may have elected to delay diagnosis.

Challenges in the Diagnosis of DS and BAG

Nonetheless, the results indicate very few studies that report rates of DS and BAG following drainage procedures for refractory gastroparesis. This is surprising as both complications have been reported for pyloroplasty when performed prophylactically to prevent gastroparesis in conjunction with procedures such as truncal vagotomy and esophagectomy for separate indications. Humphrey et al. found dumping to be more common in patients undergoing truncal vagotomy with pyloroplasty compared to patients undergoing pyloroplasty alone [45]. Brough et al. found bile acid reflux to be associated with truncal vagotomy and pyloroplasty in dogs and humans, with concurrent cholecystectomy significantly increasing symptoms [46]. The prevalence of DS after esophagectomy and gastroplasty is known to vary from 10% to 50% [21,47-50], with BAG being reported in systematic reviews of esophagectomy complications as well [51].

The underdiagnosis of DS and BAG may stem from their symptomatic overlap with other gastrointestinal conditions, coupled with the infrequent use of specific diagnostic tests for these complications. Symptoms of dumping are nonspecific and include abdominal pain, epigastric fullness, nausea, vomiting, and diarrhea [18]. These symptoms can easily be confused with other syndromes, including irritable bowel syndrome, functional dyspepsia, and gastroparesis. Post-pyloroplasty or G-POEM patients may experience dumping at higher rates than previously reported but are not diagnosed due to a lack of testing via gastric emptying scintigraphy, OGTT, or other diagnostic measures post-surgically. Similarly, BAG presents with a spectrum of symptoms, including abdominal pain, dyspepsia, nausea accompanied by bilious vomiting, a bitter taste in the mouth, decreased appetite, and heartburn, with some patients remaining asymptomatic [52]. Without diagnostic testing, these symptoms may be mistaken for other conditions such as gastroesophageal reflux disease (GERD), functional dyspepsia, or gastroparesis. Since an improvement in the Gastroparesis Cardinal Symptom Index (GCSI) score typically indicates successful treatment of gastroparesis, diagnostic tests are usually only performed when there is clinical suspicion. None of the studies included in the present review assessed the relationship between GCSI improvement and undiagnosed DS or BAG.

Limitations

This systematic review has several limitations that should be considered when interpreting its findings. The high heterogeneity among the included studies precluded the conduct of a meta-analysis and limited the generalizability of the reported findings. The available literature exhibited substantial differences in key aspects such as methodologies, study populations, interventions, and outcome measures. This diversity made direct comparisons between studies inappropriate and prevented the meaningful pooling of data for quantitative synthesis. Consequently, the review was limited to a qualitative analysis of the available evidence, focusing on narrative synthesis and descriptive summaries of the findings from individual studies. The number of studies meeting our inclusion criteria was limited, with only 10 studies included in the final analysis. This small sample size is largely due to the specificity of complications such as DS and BAG. These conditions often present with symptoms that overlap with more common gastrointestinal disorders, which means that routine testing, and consequently research specifically focusing on these complications post-surgery, is rare without prior clinical suspicion. This restricts the generalizability of our findings. Furthermore, there was an imbalance in the representation of procedures, with six studies evaluating pyloroplasty and only four evaluating G-POEM.

Inconsistent reporting of outcomes across studies further limited our analysis. While nine out of 10 studies reported on DS, only two reported on BAG. This inconsistency limits our ability to draw comprehensive conclusions about both complications. The risk of bias assessment revealed that half of the included studies had a serious risk of bias, with the remainder having moderate risk. This high risk of bias may affect the reliability of the reported outcomes and limits the strength of our conclusions. Lastly, it is important to note that none of the included studies had the primary goal of determining DS or BAG following pyloroplasty or G-POEM. This lack of focus on these complications may have led to less rigorous assessment and reporting. Further research with more standardized methodologies, definitions, and reporting is needed to draw more definitive conclusions about the incidence of DS and BAG following pyloroplasty and G-POEM for refractory gastroparesis.

## Conclusions

This systematic review highlights the paucity of data on DS and BAG following pyloroplasty and G-POEM for refractory gastroparesis. Despite the historical association of these complications with pylorus-altering procedures, our findings suggest that they are either underreported or less common in the context of treating refractory gastroparesis. The lack of comprehensive data is significant as it limits our understanding of the full spectrum of potential risks associated with these procedures, potentially affecting clinical decision-making and patient outcomes. Patients and clinicians may underestimate the risks of developing these complications, which could influence treatment choices and preparedness for managing post-procedural symptoms. Moving forward, there is a clear need for prospective studies specifically designed to evaluate the incidence of DS and BAG following these procedures and improved reporting on these complications. Such studies should employ standardized definitions, consistent diagnostic criteria, and longer follow-up periods. Additionally, routine post-procedural testing for these complications, regardless of symptom improvement, may provide a more accurate picture of their true incidence. As pyloroplasty and G-POEM continue to be utilized in treating refractory gastroparesis, a better understanding of these potential complications is crucial for informed clinical decision-making and patient counseling.

## Appendices

Appendix S1

PubMed (1971-2024 Week 22)

(G-POEM OR "Gastric peroral endoscopic myotomy" OR "Peroral endoscopic myotomy" OR "Endoscopic pyloromyotomy" OR "Endoscopic pyloric myotomy" OR "Endoscopic myotomy" OR "Endoscopic myotomy" OR "Gastrointestinal peroral endoscopic myotomy" OR "POEM procedure" OR "Gastric per-oral endoscopic myotomy" OR "Per-oral endoscopic myotomy" OR "Gastrointestinal per-oral endoscopic myotomy" OR pyloroplasty OR "Pyloric surgery" OR "Pyloric dilation" OR "Pyloric incision" OR "Pyloric release" OR "Pyloric opening" OR "Pyloric enlargement" OR "Gastric outlet surgery" OR "Pyloric dilatation" OR "plastic operation, pylorus" OR "pyloro plasty" OR "pyloroduodenotomy" OR "pyloromyoplasty" OR "pylorus plasty") AND ("Gastroparesis"[Mesh] OR Gastroparesis OR Gastropareses OR "delayed gastric emptying" OR "Gastric Stases" OR "Gastric Stasis" OR "atonia gastrica" OR "atonic stomach" OR "gastric atony" OR "gastric paralysis" OR "gastric paresis" OR "gastroplegia" OR "paralytic stomach" OR "stomach atonia" OR "stomach atony" OR "stomach paralysis" OR "stomach paresis")

Embase (1973-2024 Week 22)

('g poem' OR 'gastric peroral endoscopic myotomy'/exp OR 'gastric peroral endoscopic myotomy' OR 'peroral endoscopic myotomy'/exp OR 'peroral endoscopic myotomy' OR 'endoscopic pyloromyotomy' OR 'endoscopic pyloric myotomy' OR 'endoscopic myotomy'/exp OR 'endoscopic myotomy' OR 'gastrointestinal peroral endoscopic myotomy' OR 'poem procedure' OR 'gastric per-oral endoscopic myotomy' OR 'per-oral endoscopic myotomy' OR 'gastrointestinal per-oral endoscopic myotomy' OR 'pyloroplasty' OR 'pyloroplasty'/exp OR pyloroplasty OR 'pyloric surgery' OR 'pyloric dilation' OR 'pyloric incision' OR 'pyloric release' OR 'pyloric opening' OR 'pyloric enlargement' OR 'gastric outlet surgery' OR 'pyloric dilatation' OR 'plastic operation, pylorus'/exp OR 'plastic operation, pylorus' OR 'pyloro plasty'/exp OR 'pyloro plasty' OR 'pyloroduodenotomy'/exp OR 'pyloroduodenotomy' OR 'pyloromyoplasty'/exp OR 'pyloromyoplasty' OR 'pylorus plasty'/exp OR 'pylorus plasty') AND ('gastroparesis'/exp OR gastroparesis OR gastropareses OR 'delayed gastric emptying'/exp OR 'delayed gastric emptying' OR 'gastric stases' OR 'gastric stasis'/exp OR 'gastric stasis' OR 'atonia gastrica'/exp OR 'atonia gastrica' OR 'atonic stomach'/exp OR 'atonic stomach' OR 'gastric atony'/exp OR 'gastric atony' OR 'gastric paralysis'/exp OR 'gastric paralysis' OR 'gastric paresis'/exp OR 'gastric paresis' OR 'gastroplegia'/exp OR 'gastroplegia' OR 'paralytic stomach'/exp OR 'paralytic stomach' OR 'stomach atonia'/exp OR 'stomach atonia' OR 'stomach atony'/exp OR 'stomach atony' OR 'stomach paralysis'/exp OR 'stomach paralysis' OR 'stomach paresis'/exp OR 'stomach paresis')

*Cochrane* 

ID           Search Hits

#1           MeSH descriptor: [Gastroparesis] explode all trees     

#2           Gastroparesis OR Gastropareses OR "delayed gastric emptying" OR "Gastric Stases" OR "Gastric Stasis" OR  “atonia gastrica” OR “atonic stomach” OR “gastric atony” OR “gastric paralysis” OR “gastric paresis” OR “gastroplegia” OR “paralytic stomach” OR “stomach atonia” OR “stomach atony” OR “stomach paralysis” OR “stomach paresis”       

#3           #1 OR #2            

#4           G-POEM OR "Gastric peroral endoscopic myotomy" OR "Peroral endoscopic myotomy" OR "Endoscopic pyloromyotomy" OR "Endoscopic pyloric myotomy" OR "Endoscopic myotomy" OR "Endoscopic myotomy" OR "Gastrointestinal peroral endoscopic myotomy" OR "POEM procedure" OR "Gastric per-oral endoscopic myotomy" OR "Per-oral endoscopic myotomy" OR "Gastrointestinal per-oral endoscopic myotomy" OR pyloroplasty OR "Pyloric surgery" OR "Pyloric dilation" OR "Pyloric incision" OR "Pyloric release" OR "Pyloric opening" OR "Pyloric enlargement"  OR "Gastric outlet surgery" OR "Pyloric dilatation" OR “plastic operation, pylorus” OR “pyloro plasty” OR “pyloroduodenotomy” OR “pyloromyoplasty” OR “pylorus plasty”    

#5           #3 AND #4         

Web of Science

G-POEM OR "Gastric peroral endoscopic myotomy" OR "Peroral endoscopic myotomy" OR "Endoscopic pyloromyotomy" OR "Endoscopic pyloric myotomy" OR "Endoscopic myotomy" OR "Endoscopic myotomy" OR "Gastrointestinal peroral endoscopic myotomy" OR "POEM procedure" OR "Gastric per-oral endoscopic myotomy" OR "Per-oral endoscopic myotomy" OR "Gastrointestinal per-oral endoscopic myotomy" OR pyloroplasty OR "Pyloric surgery" OR "Pyloric dilation" OR "Pyloric incision" OR "Pyloric release" OR "Pyloric opening" OR "Pyloric enlargement" OR "Gastric outlet surgery" OR "Pyloric dilatation" OR "plastic operation, pylorus" OR "pyloro plasty" OR "pyloroduodenotomy" OR "pyloromyoplasty" OR "pylorus plasty" (All Fields) and Gastroparesis OR Gastropareses OR "delayed gastric emptying" OR "Gastric Stases" OR "Gastric Stasis" OR "atonia gastrica" OR "atonic stomach" OR "gastric atony" OR "gastric paralysis" OR "gastric paresis" OR "gastroplegia" OR "paralytic stomach" OR "stomach atonia" OR "stomach atony" OR "stomach paralysis" OR "stomach paresis" (All Fields)
